# Docosahexaenoic Acid Supplementation Early in Pregnancy May Prevent Deep Placentation Disorders

**DOI:** 10.1155/2014/526895

**Published:** 2014-06-12

**Authors:** Jorge A. Carvajal

**Affiliations:** ^1^Unidad de Medicina Materno Fetal, División de Obstetricia y Ginecología, Escuela de Medicina, Facultad de Medicina, Pontificia Universidad Católica de Chile, 8330024 Santiago, Chile; ^2^Centro de Investigaciones Médicas, Pontificia Universidad Católica de Chile, Marcoleta 391, 8330024 Santiago, Chile

## Abstract

Uteroplacental ischemia may cause preterm birth, either due to preterm labor, preterm premature rupture of membranes, or medical indication (in the presence of preeclampsia or fetal growth restriction). Uteroplacental ischemia is the product of defective deep placentation, a failure of invasion, and transformation of the spiral arteries by the trophoblast. The failure of normal placentation generates a series of clinical abnormalities nowadays called “deep placentation disorders”; they include preeclampsia, fetal growth restriction, preterm labor, preterm premature rupture of membranes, in utero fetal death, and placental abruption. Early reports suggested that a LC-PUFAs (long chain polyunsaturated fatty acids) rich diet reduces the incidence of deep placentation disorders. Recent randomized controlled trials are inconsistent to show the benefit of docosahexaenoic acid (DHA) supplementation during pregnancy to prevent deep placentation disorders, but most of them showed that DHA supplementation was associated with lower risk of early preterm birth. We postulate that DHA supplementation, early in pregnancy, may reduce the incidence of deep placentation disorders. If our hypothesis is correct, DHA supplementation, early in pregnancy, will become a safe and effective strategy for primary prevention of highly relevant pregnancy diseases, such as preterm birth, preeclampsia, and fetal growth restriction.

## 1. Introduction

Preterm birth is the one that occurs after 22 and before 37 weeks of gestation [1]. Its incidence ranges from 8 to 10% of all births, although with significant regional variations, with an incidence as high as 10–12% in the US, or as low as 5% in Chile and the European Union [2]. Excluding congenital malformations, 75% of perinatal deaths and 50% of childhood neurological disabilities are directly attributable to prematurity [2].

Preterm birth can be originated by (1) preterm labor, (2) preterm premature rupture of membranes, and (3) indicated preterm birth (premature medical termination of pregnancy due to maternal or fetal problems) [2–4]. Each of these three groups corresponds roughly to a third of all premature births; the data generated in our center confirms this universal trend [5]. Although many etiologies underlie any of these clinical groups, uteroplacental ischemia originated in disorders of deep placentation may be a common cause [6–13].

## 2. Uteroplacental Ischemia and Preterm Birth

An emerging hypothesis correlates by etiology idiopathic preterm labor with the phenomena of reduced blood flow to the uterus and placenta: uteroplacental ischemia. This hypothesis is supported by clinical and experimental evidence; for example, pregnant women at high altitude (>4,000 meters above sea level) have triple chance of premature delivery than women living at sea level (12 versus 4%) [14]. Clinical conditions that are a reflection of placental ischemia, such as preeclampsia and fetal growth restriction, are frequently associated with premature onset of labor [5, 15]. The anatomical-clinical correlation between preterm birth and placental morphology (histology) indicating uteroplacental ischemia has also been demonstrated [7, 16–18].

Decreased uteroplacental blood flow can be estimated by studying vascular resistance in the uterine artery by Doppler. In pregnant women without risk factors for preterm labor, increased vascular resistance in the uterine artery increases by 5 times the risk of preterm birth [19]. Moreover, in women in spontaneous preterm labor, increased vascular resistance in the uterine arteries is associated with double risk of premature birth [20–22].

The first clinical series, investigating the different etiologies of preterm labor in 50 women, including placental histology and uterine artery Doppler velocimetry, concluded that 30% of patients with preterm labor show uteroplacental ischemia [17]. Our group conducted a controlled clinical trial in a group of 145 patients admitted to our hospital in preterm labor. In each of these patients and in a control group, we assessed the presence of uteroplacental ischemia (uterine artery Doppler, birth weight, placental histology, and placental weight) and infection (placental histology and amniotic fluid cultures). We reported that 30% of patients with idiopathic preterm labor have clinical and/or laboratory evidences of uteroplacental ischemia [7]. The group with uteroplacental ischemia as the etiology of preterm birth has larger neonatal morbidity than the group with neither infection nor ischemia [7].

Our group proposed that ischemia in any of the components of the uteroplacental unit (trophoblast, decidua, fetal membranes, or myometrium) generates paracrine mediators that trigger the premature onset of myometrial contractile activity. We have reported the role of fetal membranes derived B-type natriuretic peptide (BNP) in maintaining myometrial quiescence during pregnancy, and that the premature decrease of BNP production may cause preterm labor and preterm birth [23–25]. We postulate that uteroplacental ischemia, induced by abnormal placentation, may produce a premature decline in BNP production, being responsible for the premature activation of the myometrium. We studied the effect of hypoxia on BNP production by trophoblast explants; we found that hypoxia decreases by 50% the BNP production (unpublished).

To completely understand uteroplacental ischemia as a cause of premature birth, the series of events between ischemia and increased myometrial contractility remain to be determined. However what causes uteroplacental ischemia is already known: defective deep placentation.

## 3. Deep Placentation

Implantation and placentation establish a physical contact of the embryo/fetus and the mother, with two main objectives: (A) to establish a structural support of the embryo to the uterus and (B) to bring maternal and fetal circulation close enough to allow an adequate transfer of gases, nutrients, and waste products. Placentation begins with the implantation of the blastocyst; the outermost cells of the blastocyst (extra-embryonic cells) give rise to the trophoblast, a specialized epithelium that during implantation invades the decidua (maternal tissue originated in the endometrium prepared to receive the embryo) and the inner myometrium, developing the placenta. The embryoblast is surrounded by a “trophoblastic shell” of syncytiotrophoblast, through which columns of proliferating cytotrophoblast penetrate to invade the uterine stroma. Cytotrophoblast that penetrates beyond the syncytiotrophoblast shell assumes the extravillous cytotrophoblast lineage. The trophoblast forms two types of chorionic villi: floating and anchoring. The floating villi occupy the intervillous space (“gaps” filled with maternal blood) allowing the transport of gases and nutrients. Anchoring villi penetrate the uterine wall to provide physical support to the fetus and ensure adequate placental perfusion [26]. The columns of trophoblast are penetrated by extra-embryonic mesoderm, in which fetoplacental blood vessels form by vasculogenesis, contributing to placental formation [27].

During the process of placentation, trophoblast cells attach to the basal membrane surrounding the stroma of these two types of villi. In the villi, the trophoblast cells fuse to create an external layer called syncytiotrophoblast; at the distal end of the anchoring villi, the trophoblast breaks the basal membrane and forms “trophoblast cell columns.” The cell columns are formed by a subpopulation of cytotrophoblast cells called extravillous trophoblast that proliferates, invades the decidua and superficial layer of myometrium, and transforms the spiral arteries (a terminal branch of the uterine arteries that reach the endometrial surface) [26, 28]. Complete transformation of spiral arteries is required for a successful pregnancy since the transformed spiral arteries become low resistance vessels allowing a normal blood flow to the fetoplacental unit [10].

The mechanisms underlying extravillous trophoblast proliferation and invasion have not been fully established, but it is known that many molecular pathways are involved: (a) cellular interaction systems, whether cell-cell (cadherins) or cell-extracellular matrix (integrins), (b) proteolysis systems such as urokinase plasminogen activator (uPA)/plasminogen activator inhibitor type-2 (PAI-2) and matrix metalloproteinase type 9 (MMP-9)/tissue inhibitor of metalloproteinase-3 (TIMP-3), and (c) growth factors/vascular growth factors such as insulin growth factor II (IGF II) and its binding protein-1 (IGFBP-1), vascular endothelial growth factor (VEGF) and its receptors (Flt-1), and transforming growth factor beta (TGF*β*) and its receptor (endoglin) among others [28–30].

The trophoblast-associated remodeling of the spiral arteries is a process of profound changes of the arterial wall of these vessels, mainly characterized by (a) replacement of the vessel wall (media and endothelium) by endovascular trophoblast and (b) replacement of the muscular and elastic arterial wall by interstitial trophoblast and fibrinoid material [31–34]. To transform the spiral arteries, the trophoblast invades the maternal tissue (decidua, myometrium, and arteries) via two different routes: interstitial and endovascular. In the interstitial invasion, the trophoblastic cells migrate towards the arterial wall replacing the musculoelastic wall [31, 32]. The interstitial trophoblast may also be originated from extravasation of the intraluminal trophoblast [34]. In the endovascular invasion the endovascular trophoblastic cells infiltrate the lumens and walls of the arteries replacing the endothelium [31, 32, 34]; the endovascular trophoblast results from intravasation of interstitial trophoblast or by intraluminal migration of trophoblast [34].

The molecular basis of trophoblast-associated remodeling of the spiral arteries have not been fully established but integrins such as *α*v*β*3, *α*1*β*1, and VE-cadherin (vascular endothelial cadherin); adhesion molecules like VCAM type-1 (vascular endothelial cell adhesion molecule 1), PECAM- 1 (platelet cell adhesion molecule 1), and NCAM-1 (nerve cell adhesion molecule 1); and protease/anti-type protease systems such as MMP-9 and PAI-1/PAI-2 play a key role [35–40]. In knockout models for these factors, the more clearly linked to a severe abnormality of placentation is the *α*V-*β*3 integrin knockout mouse; *α*V-*β*3 integrin deficiency is associated with a 50% reduction in litter size and fetal mortality of 10% and is accompanied by a clear reduction in placental perfusion [35]. PAI-1 is produced by endothelial cells and is a marker of endothelial activation; PAI-1 level increases during pregnancy, but the increase is larger in the presence of endothelial dysfunction and abnormal placentation [41]. PAI-2 is produced by the cytotrophoblast; its production increases in normal pregnancy but is reduced in abnormal placentation [42]. In normal pregnancy PAI-1/PAI-2 reason is progressively reduced with increasing placental mass, but in the presence of abnormal placentation the PAI-1/PAI-2 ratio has been used as a marker of placental insufficiency and high risk of developing preeclampsia or fetal growth restriction [43–47].

A key question is how the trophoblast invasion is directed to the blood vessel to proceed to its transformation. Attention has been focused on two areas: (a) biological effects of oxygen partial pressure on the trophoblast and (b) maternal endothelium-derived factors. Oxygen partial pressure: it has been suggested that extravillous trophoblast proliferation and invasion is modulated by the interstitial partial pressure of oxygen [34]. At low partial pressures (2% O_2_), as is the case at the start of the trophoblast cell column, the trophoblast proliferates intensively but does not express the integrins *α*v*β*3 and *α*1*β*1, needed for invasion. In the presence of higher oxygen partial pressures (8% O_2_, closer to the blood vessel) the trophoblast exhibits an invasive phenotype, showing that oxygen modifies cell proliferation and differentiation [48]. The underlying mechanism involves the transcription factor HIF-1*α* (hypoxia inducible factor-1*α*) that modulates the secretion of transforming growth factor *β*3 (TGF*β*3), *α*1*β*1 integrin expression, and production of MMP-9 [49]. Endothelium-derived factors: they play a primary role in (a) trophoblast chemotaxis, (b) trophoblast primary interaction with the endothelium (adhesion molecules), and (c) maintenance of arteriolar vasodilation [50–53]. It is known that in vitro VEGF plays a chemotactic role in trophoblast migration [53]; VEGF and PGF (placental growth factor) are produced by the cyto- and syncytiotrophoblast and their secretion is modulated by hypoxia (VEGF increase and PGF decrease in response to hypoxia) [52]. VEGF upregulates the production by endothelial cells of proinvasive integrins (*α*v*β*3 and *α*1*β*1) and upregulates the expression of the intracellular adhesion molecule-1 (ICAM-1) that participates in the process of trophoblast-endothelium interaction, a key event in vascular transformation [50, 51].

## 4. Timing of Trophoblast Invasion and Spiral Arteries Transformation

The classical view is that this process occurs in two stages: (1) the transformation of the decidual segment of the spiral arteries by a “first wave” of endovascular trophoblast migration in the first trimester; and (2) the transformation of the myometrial segments of the spiral arteries in the second trimester by a “second wave” of trophoblast [54]. However, more recent studies have suggested that trophoblast invasion of the spiral arteries is a continuous process [55]. Anyway, deep transformation of the spiral arteries, reaching the myometrial segment of the spiral arteries, is required for successful placentation and normal pregnancy progression [10] while deep placentation failure may cause clinically relevant pregnancy disorders [6]. The earlier in pregnancy impaired placentation starts, the larger deep placentation defect and the greater clinical consequences will be observed. Similarly, those interventions to improve placentation will be more successful the earlier in pregnancy they are started [56, 57].

## 5. Defective Deep Placentation and Pregnancy Diseases

The failure of normal placentation generates a series of clinical abnormalities nowadays called “deep placentation disorders” [8, 10, 31]. Originally deep placentation disorders were considered preeclampsia (PE) and fetal growth restriction (FGR) [6]; however, it has been shown that deep placentation disorders also included preterm labor (PL) and preterm premature rupture of membranes (PPROM), in utero fetal death and placental abruption [9, 11, 12]. Considering that all these deep placentation disorders are the main problems of modern obstetrics, generating enormous adverse impact on maternal and perinatal health, great efforts have been invested in strategies to prevent deep placentation disorders [58–64]. We should observe that most of these strategies are based on secondary prevention, that is, prevention of recurrence, guiding prediction strategies, and/or treatments to high risk pregnancies.

Some of the strategies to prevent deep placentation disorders have shown benefit: aspirin for prevention of PE and FGR [65, 66] or the use of progesterone for prevention of PL and PPROM [67]. It is important to observe that the preventive effect is stronger when the intervention starts earlier in pregnancy [68, 69]. Probably the need of using these drugs early in pregnancy reflects the effect they have on improving placentation, avoiding deep placentation disorders [6]. Since these strategies to prevent deep placentation disorders are aimed to high risk pregnancies, we observe two problems: an early identification of population at risk is required (to start treatment) and the opportunity for primary prevention is lost (low-risk population is not considered). Strategies of deep placentation disorders prevention are required to implement at the population level, that is, drugs or supplements that can be administered to the general population to prevent the occurrence of deep placentation disorders (primary prevention); unfortunately studies of primary prevention have failed to be effective [70–72].

## 6. Polyunsaturated Fatty Acids

**Figure 1:**
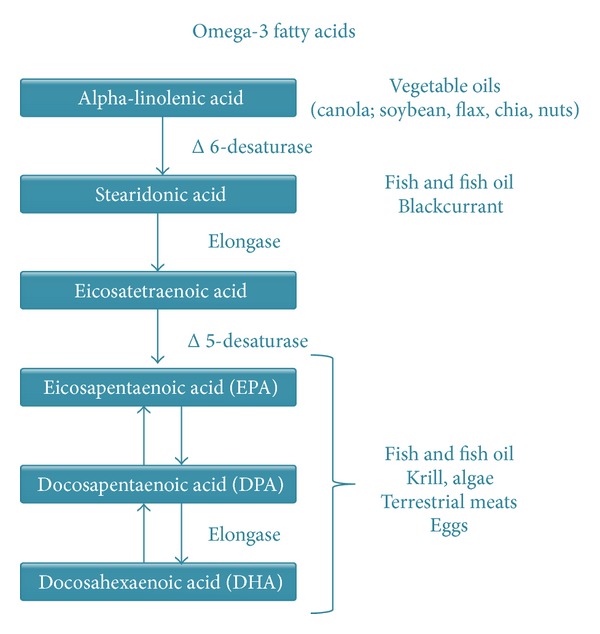
Docosahexaenoic acid (DHA) is an essential fatty acid of the family of long chain polyunsaturated fatty acids (LC-PUFAs) or omega-3 fatty acids. In humans, DHA can be consumed (mainly fish or fish oil) or converted from EPA.

Docosahexaenoic acid (DHA) is an essential fatty acid of the family of long chain polyunsaturated fatty acids (LC-PUFAs) or omega-3 fatty acids [73]. To this family eicosapentaenoic acid (EPA) and alpha-linolenic acid (*α*-LA) also belong (Figure 1). These fatty acids are considered essential because our body cannot synthesize them; thus the LC-PUFAs must be acquired through food [74, 75]. The LC-PUFAs are essential components of phospholipids present in all our tissues and actively involved in the functional regulation of cellular and subcellular membranes [74–76]. Early reports, mainly observational, suggested that LC-PUFAs rich diet reduces the incidence of deep placentation disorders (preeclampsia, intrauterine growth restriction, and preterm delivery) [77–79]. We recently submitted a critical review of the safety and efficacy of DHA supplementation during pregnancy to improve pregnancy outcome in general population of pregnant women; we conclude that the evidence currently available does not support or completely rule out this intervention during pregnancy [80].

## 7. Effect of DHA Supplementation on Pregnancy Duration

Three recent randomized studies have reported the effect of DHA supplementation on pregnancy duration in general population of pregnant women: one of them showed a significant reduction in the proportion of preterm birth before 34 weeks (1.09% versus 2.25%, DHA versus placebo, resp., RR 0.49, 95% CI 0.25–0.94, *P* = 0.03) [81]. The second study did not find differences in gestational age at delivery (39.1 ± 1.7 versus 39.0 ± 1.9 weeks, DHA versus placebo, resp., mean ± standard deviation, *P* > 0.05) or the proportion birth before 37 weeks (10.1% versus 8.3%, DHA versus placebo, resp., RR 1.2, 95% CI 0.8–1.8, *P* = 0.33) [82]. Finally, a more recent study demonstrated a significant prolongation of pregnancy and a reduction in the rate of delivery before 34 weeks (0.6% versus 4.8%, *P* < 0.05, DHA versus placebo, resp.) [83]. A randomized controlled trial in high risk population (women with a history of prior spontaneous singleton preterm birth) failed to demonstrated benefit of DHA supplementation to prevent preterm birth (37.8% versus 41.6%; RR 0.91, 95% CI 0.77–1.07, DHA versus placebo, resp.) [84]. The systematic Cochrane review studying the effect of marine oil (70% DHA; 30% EPA) supplementation during pregnancy failed to demonstrate a significant reduction of preterm birth but suggested a significant reduction of birth before 34 weeks (RR 0.69, 95% CI 0.49–0.99), including two trials [85]. A recent meta-analysis of LC-PUFAs supplementation during pregnancy shows that women receiving LC-PUFA had a 26% lower risk of early preterm delivery (<34 weeks) (RR = 0.74; 95% CI 0.58–0.94) and no difference in the risk of preterm delivery (<37 weeks) (RR = 0.91; 95% CI 0.82–1.01) [86]. Because of the heterogeneity of the studies and the great variability in the observational studies, they conclude that more studies are needed to confirm the findings, especially in undeveloped countries [86].

## 8. Effect of DHA Supplementation on Birth Weight

The newborn weight was presented as a secondary analysis of two randomized studies [81, 82] showing that average birth weight significantly increased in women who received DHA. The first study reported a lower proportion of infants weighing less than 2500 g in the group of women receiving DHA supplementation compared to the control group (3.41% versus 5.27%, resp., RR 0.65, 95% CI 0.44–0.96, *P* = 0.03) [81]. The other study showed that DHA supplementation reduced the risk of intrauterine growth restriction (weight below the 10th percentile for gestational age), only in primiparous women (7.1% versus 14%, DHA versus placebo, resp., RR 0.5, 95% CI 0.3–1.0, *P* = 0.03) [82]. The meta-analysis failed to demonstrate a reduction in the rate of low birth weight in response to LC-PUFAs supplementation [85–87].

## 9. Effect of DHA Supplementation on the Incidence of Preeclampsia

Observational studies have suggested that increased intake of polyunsaturated fatty acids reduces the risk of preeclampsia [88–90]. Furthermore lower concentration of polyunsaturated fatty acids has been shown in placentas of women with PE compared with normotensive pregnant women [91], in addition to an inverse relationship between LC-PUFAs and sFLT-1 (a marker of defective vasculogenesis) in patients with PE [92, 93]. However, two meta-analyses failed to demonstrate reduced risk of PE associated with higher intake of LC-PUFAs [86, 87] and the same goes for the systematic review of marine oil [85]; these two meta-analyses do not differentiate between early or late PE.

## 10. Polyunsaturated Fatty Acids and Placentation

The effect of fatty acids in extravillous trophoblast cell lines (HTR8/SVneo cells) has been studied, showing that DHA, EPA, and AA (arachidonic acid) have a proangiogenic effect, expressed by an increase in the development of capillary sprouts [94]. In the same model, it has been demonstrated that linoleic acid supplementation in vitro stimulates the production of proangiogenic factors and the formation of capillary sprouts [95]. The comparative analysis showed that DHA is more powerful in stimulating extravillous trophoblast vasculogenesis [94, 96].

Another line of research using immortalized trophoblast cells of neoplastic origin (BeWo cells) demonstrated that DHA reduces oxidative stress [97]. Similarly, DHA supplementation during pregnancy in the rat reduces placental oxidative stress [98]. This is important since placental oxidative stress may play a key role in the pathophysiology of deep placentation disorders [99]. Also, defective deep placentation induces oxidative stress that may cause the clinical disorders related to defective placentation [100, 101].

Additionally, DHA supplementation is associated with elevated markers of trophoblast proliferation, measured in placentas obtained at term of pregnancy [102]. The direct effect of DHA supplementation during pregnancy on placentation, for example, using placental bed biopsies, has not been studied. However, in the rat it was shown that LC-PUFAs supplementation early in pregnancy increased fetal and placental size [98].

## 11. Conclusions

Considering these antecedents we propose that maternal supplementation with LC-PUFAs, especially DHA, reduces deep placentation disorders, by improving deep placentation. We consider that more information is needed to get a final conclusion about the therapeutic role of DHA supplementation during pregnancy; we have recently obtained ethical approval and financial support for a randomized placebo controlled trial of DHA supplementation from early pregnancy to prevent deep placentation disorders.

We need such a new trial, since there are still doubts about the efficacy of DHA to improve perinatal outcome. The difference among studies may be explained, at least partially, by the timing of supplementation and the main outcome measured. Regarding the timing of supplementation we postulate that the effect of DHA, which improves the clinical outcome, is to prevent defective deep placentation. Therefore, the more early in pregnancy DHA starts, the greater the effect on improving placentation is, making it possible to achieve a significant improvement in clinical outcomes. In fact, many of the studies failing to demonstrate the effect of DHA started supplementation after 16 weeks of pregnancy [82, 84]. Concerning the main outcome of the study, we must observe that early preterm (<34 weeks) and late preterm (>36 weeks) are probably two different diseases with different etiologies; the same happens for early and late preeclampsia and early or late fetal growth restriction [103–105]. Current evidence suggests that early prematurity (preterm labor or preterm premature rupture of membranes), early fetal growth restriction, and early preeclampsia share deep placentation defects as common etiology [6]. Thus, deep placentation disorders are the main outcome perhaps clinically modifiable with DHA supplementation early in pregnancy.

LC-PUFAs, including DHA, supplementation in pregnant women, breastfeeding mothers, and infants, have been well tolerated (more than 90% adherence to the protocol) and did not generate any serious adverse events [81, 83, 85–87]. Minor potential side effects are nausea, intestinal gas, bruising, and prolonged bleeding, fishy taste, belching, nosebleeds, and loose stools. Taking DHA with meals can often decrease these side effects [81, 83, 106]. Consuming large amounts of fish oil from some dietary sources is possibly unsafe. Some fish meats (especially shark, king mackerel, and farm-raised salmon) can be contaminated with mercury and other industrial and environmental chemicals, but fish oil supplements typically do not contain these contaminants [107, 108]. The maternal intake of LC-PUFA during pregnancy and lactation is considered very important, since the LC-PUFAs are provided during perinatal development through placental transfer and maternal milk [80, 109]; thus DHA or LC-PUFAs intake during pregnancy is encouraged [110].

Although a precise DHA intake level for pregnant women has not been fully established [111], the worldwide recommendation for DHA intake during pregnancy is 200 mg/day [112]. In Chile there is not a local recommendation, but powdered milk supplied to pregnant women in the public health system (*Purita Mamá*) contains DHA 240 mg + EPA 76 mg per 100 g; this taken to 200 mL provides 60 mg DHA and 19 mg of EPA per day. Beyond the nutritional recommendation (eating fish), and the contribution of milk, in Chile there is no national standard for the indication of DHA in capsules during pregnancy. While there are products on the market containing DHA with other vitamins, for pregnant women, their use is not a regular practice among obstetricians in our country. Thus, it seems possible and ethically plausible to conduct a randomized study in which one arm of the study received a placebo containing no DHA.

Most of the intervention trials have included few participants and provided fish oil supplements that contain large and varying doses of DHA (100 mg to 5 gr/day) [106]. The two trials that demonstrate benefit of DHA supplementation to prevent preterm birth use either 600 [83] or 800 mg DHA per day [81]; thus a new trial must use a dose of 600 or 800 mg DHA per day.

Studies have also measured cognitive development or visual accuracy of children in the long term [113–118]. Thus, children monitoring, about visual or neurocognitive development, would be also important in a clinical DHA trial.
